# Emergency physicians’ perceptions of critical appraisal skills: a qualitative study

**DOI:** 10.1186/s12909-022-03358-y

**Published:** 2022-04-15

**Authors:** Sumintra Wood, Jacqueline Paulis, Angela Chen

**Affiliations:** 1 Department of Emergency Medicine, Maimonides Medical Center, 4802 10th Avenue, Brooklyn, NY 11219 USA; 2 Department of Emergency Medicine, New York University School of Medicine, New York, NY USA; 3 Department of Emergency Medicine, Icahn School of Medicine at Mount Sinai, New York, NY USA

**Keywords:** Critical appraisal, Medical education, Qualitative research

## Abstract

**Background:**

Critical appraisal of medical research is a valuable skill set that emergency physicians must learn in order to become competent clinicians. Despite the need for effective critical appraisal skills training, these skills have remained difficult to teach and assess. This study aimed to explore emergency physicians’ perceptions of the barriers and motivations for learning critical appraisal skills in order to develop more successful critical appraisal training methods for Emergency Medicine (EM) residents.

**Methods:**

This qualitative study involved in-depth, semi-structured interviews with emergency physicians interested in education and administration at an urban academic hospital. Transcribed interviews were descriptively coded by three main reviewers. A coding template was developed after coding an initial set of interviews and used to code the remaining transcripts. A thematic analysis of the codes was conducted to create a summary report which was given to the interviewees as part of a member checking process to further solidify themes.

**Results:**

Fourteen emergency physicians participated in the study. They described time limitations, perceived difficulty, and disinterest as major barriers to learning critical appraisal. Physicians noted patient care as well as professional identity goals of being a good educator or researcher as motivations for developing critical appraisal skills.

**Conclusion:**

There remain significant challenges to learning critical appraisal skills as well as an increasing need to build these skills during residency. Educational theories and a greater emphasis on professional identity formation during residency can be incorporated to create a more effective approach to teaching critical appraisal skills despite these barriers.

**Supplementary Information:**

The online version contains supplementary material available at 10.1186/s12909-022-03358-y.

## Background

Critical appraisal of medical research is an increasingly important skill set that physicians in-training must achieve in order to become competent clinicians [1, 2]. The Accreditation Council for Graduate Medical Education (ACGME) requires that Emergency Medicine (EM) residents learn how to critically appraise the literature and apply evidence-based medicine to their practice [3]. Beyond training requirements, it is important that emergency physicians maintain strong critical appraisal skills in order to deliver high quality medical care to their patients and educate future emergency physicians. A previous survey assessing the broader category of evidence based medicine (EBM) practice within EM residency programs noted limited curriculum time given to EBM training, inadequate faculty training and faculty apathy as barriers to teaching EBM. Despite acknowledging the value of EBM, there was significant variability in terms of EBM curricula and faculty support among EM residency programs [4].

Multiple studies have also attempted to teach critical appraisal skills, or the broader category of EBM, to both residents and attending physicians, but with varying degrees of success [5–8]. Furthermore, accurately assessing residents’ critical appraisal skills is a difficult task, as these concepts cannot be easily evaluated and may require more than one educational approach to enhance residents’ learning [9]. Along with these challenges, EM residents may not be as motivated to learn critical appraisal skills given the required demands of learning a variety of procedural skills and medical knowledge related to multiple different specialties in a short period of time.

Despite a clear need for effective critical appraisal skills training for emergency physicians, there is as of yet no standard, comprehensive critical appraisal program [4, 10]. In order to develop more effective critical appraisal skills training methods for EM residents it is important to first understand emergency physicians’ perceptions of critical appraisal skills.

We conducted a qualitative research study to answer the following research questions:

1. What are emergency physicians’ expectations and prior knowledge of critical appraisal skills?
2. What are the facilitators and barriers to critical appraisal skills for different stage learners?
3. What motivates emergency physicians to learn and maintain critical appraisal skills?

The following theories of motivation and adult learning served as conceptual frameworks for our study: social cognitive theory, cognitive load theory, and self-determination theory [11].

By enhancing our understanding of critical appraisal from a qualitative perspective, this study aimed to develop a more nuanced and accurate educational model for continued emergency physician training, with the ultimate goal of using these skills to provide safer patient care.

## Methods

### Setting

This was a single institution study conducted at an emergency department (ED) in an urban tertiary care center affiliated with a 3-year accredited ACGME EM residency program with 16 residents per year (48 total), as well as fellows in the sub-specialties of Medical Education, Simulation, and Ultrasound.

### Participants

This study was conducted from November 2018 to April 2019 and included faculty and residents in the department of Emergency Medicine (see Table 1). Purposive sampling was used to obtain a diverse group of 14 total interviewees. There was no financial incentive for participation in the study, and initial consent was obtained via email, and then again at the start of the interview.

**Table 1:** Participants

Gender	Years post-residency
M	20
M	16
M	16
M	15
M	12
F	5
F	5
F	3
M	3
M	2
F	2
F	0 (resident)
M	0 (resident)
F	0 (resident)

Participants in study and their years practicing EM

### Design instrument

Our study incorporated a constructionist approach to grounded theory as described by Charmaz, which acknowledges the social and constructed nature of reality as well as the importance of the interactions between the researchers and interviewees in co-constructing the data [12].

**Fig. 1:**
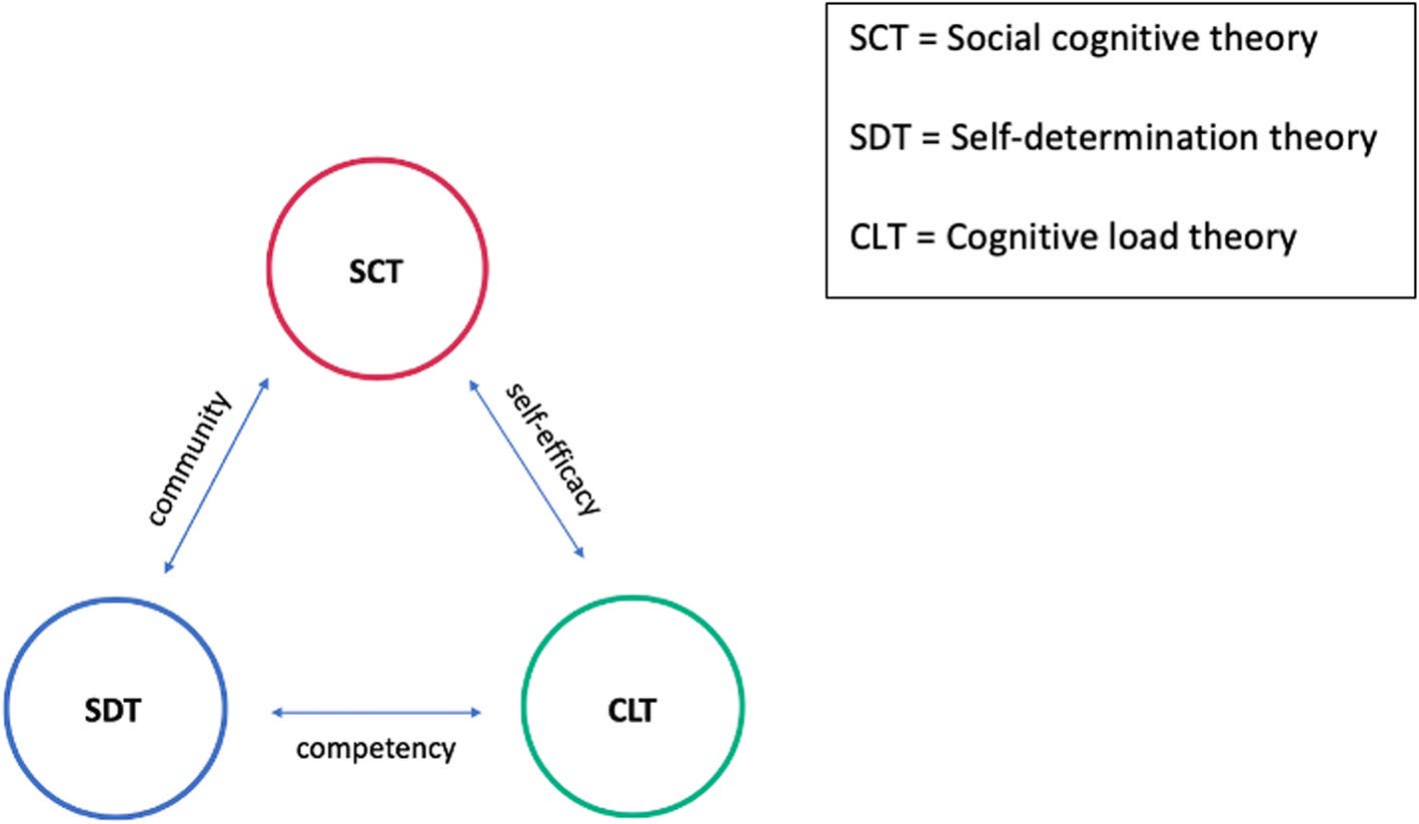
The interview guide was developed using theories of motivation and adult learning. Self-determination theory, social cognitive theory, and cognitive load theory all interrelate as theories that explain humans’ intrinsic motivation for learning. SDT emphasizes humans need to relate to community and strive for competency, whereas SCT describes the influence of community on human motivation and sense of self-efficacy. CLT supports ways in which humans can develop competency and self-efficacy

We developed our interview guide using theories of motivation and adult learning (Fig. 1). Self-determination theory (SDT) provides an underlying explanation for humans’ intrinsic motivation for learning [13]. The primary factors that drive intrinsic motivation are humans’ basic needs for autonomy, competence, and relatedness to their community. Our study looked to evaluate how emergency physicians work to develop autonomy, achieve competence, and build a sense of community in regards to critical appraisal skills.

Additional theories on motivation also contributed to the design of our study, including social cognitive theory (SCT) and cognitive load theory (CLT). SCT states that people are motivated due to their interactions within social environments [14]. Our study investigated the ways in which emergency physicians feel their social environment influences the development of critical appraisal skills.

CLT provides a way of understanding how the effort to perform a task can influence trainees’ learning and memory. The focus of CLT is to develop strategies to decrease extraneous load, adjust intrinsic load to correspond with trainee level, and optimize germane load [15]. More targeted questioning during interviews focused on barriers that emergency physicians face when learning critical appraisal.

Given the influence of different aspects of SDT, SCT, and CLT on adult learning, the interview guide questions (Additional file 1: Appendix A) were developed to address these distinct components and how they could interact to impact the development of critical appraisal skills. The interview guide questions were reviewed by a panel of emergency physicians with backgrounds in education and administration as well as the research team and revised based on their feedback prior to conducting the interviews.

Two emergency physicians (AC, JP) with no work affiliation with the hospital site conducted the interviews. They each received training in the interview process prior to performing the interviews and used the interview guide to keep the interview structures similar, but were allowed to add additional supporting questions or change the order in which the questions were asked. The two interviewers along with a single investigator (SW) coded all transcripts separately and met at intervals to reach consensus about codes and themes. Analyst triangulation was performed by having two expert independent coders review a subset of the interview transcripts to enhance credibility [16].

Interviews occurred either in person or via phone, and lasted generally 30–60 min. All interviews were recorded, de-identified, and transcribed verbatim using an outside transcription service. The original recordings were destroyed and the transcripts kept securely in one location to preserve confidentiality. Informed consent was obtained for all physicians involved in the interview process for both the interview and recording.

### Data analysis

Careful coding as well as memo-writing to promote connections of concepts and interpretation of the data helped to structure the data analysis process. Initial codes were created based on key words and concepts that were found in common throughout the initial 8 interview transcripts and then reviewed with the research team to create an initial coding template. The initial code template was used to code the remaining 6 interview transcripts. After completing coding of all 14 transcripts the codes were discussed again as a research team with revisions and new codes incorporated as agreed upon by the team. Each member of the research team contributed to the coding analysis of all transcripts and reviewed the final code template for credibility (Additional file 2: Appendix B). Coding of all interview transcripts was conducted using Dedoose software version 8.2.14.

All interviewees were given a summary report of the key emerging themes of the study along with descriptive interview data quotes as part of a synthesized member checking (SMC) process [17]. Interviewees were encouraged to provide any additional commentary in response to the report. These responses were then reviewed by the research team and contributed to the final analysis of the key themes as a method to improve the credibility of the results [17].

## Results

The analysis of the interviews revealed multiple themes regarding emergency physicians’ perceptions of the difficulties and strengths of critical appraisal, as well as strategies of how to motivate residents to learn and maintain critical appraisal skills based on their own experiences.

At the start of the interview, each physician was asked to define critical appraisal in their own terms. Interviewees described critical appraisal as “A structural approach to assessing, analyzing, and making your own conclusion with respect to an article in a journal, book that you read based on the preset set of criteria.” (Male, 16 years post-residency).

Major themes identified during the interview analysis included: 1. Barriers to learning critical appraisal 2. Importance of critical appraisal 3. Motivation for critical appraisal 4. Facilitating engagement with critical appraisal 5. Teaching critical appraisal 6. Level of competency with critical appraisal.

1. Barriers to learning critical appraisal

Interviewees described three main problematic aspects of learning critical appraisal skills: lack of interest or relevance, time limitations, and perceived difficulty to learn these skills. In combination, these barriers make it difficult for emergency physicians, especially trainees, to learn critical appraisal skills. “It’s a skill that has nothing to do or very little to do with clinical medicine. It’s a very significant amount of knowledge to understand and requires – on some level, it’s like learning a new language, and it just requires a significant investment of time that most physicians are not willing to undertake...” (Male, 12 years post-residency).

a) Lack of interest or relevance.

One main difficulty that many interviewees described was the boring nature of critical appraisal in itself. One physician stated that critical appraisal was “a dry topic and doesn’t engage the learners, especially someone who is young …” (Male, 16 years post-residency). Similar to the idea of critical appraisal being uninteresting was the concept that these skills feel somewhat out of the scope of what most emergency residents want to learn during their training.

b) Time limitations.

Residents noted the difficulty in trying to learn critical appraisal skills on top of the core EM knowledge they are required to learn, stating, “I think it’s hard especially as a resident to just be focusing on even just learning the literature.” (Female, resident). Attending physicians involved in education also felt that there was limited time to include critical appraisal skills training within weekly conference didactics.

c) Perceived difficulty.

Critical appraisal skills were seen as difficult to understand compared with learning the more practical knowledge of how to diagnose and treat medical emergencies. Residents noted that “most people don’t like doing things they’re not good at, right” and also emphasized that “It sometimes feels like you won’t ever reach that expert level so you just kind of give up.” (Female, resident).

Attending physicians noted that it could be hard to learn critical appraisal skills because of the challenges of understanding research methodology as well as the sheer amount of medical literature available to review.

2. Importance of critical appraisal

Despite these barriers, all interviewees described the value of learning critical appraisal skills. Attendings noted the increasing importance of evidence-based medicine to guide physicians’ practice as a reason for all physicians to develop and maintain their critical appraisal skills.

“We’re moving further and further away from eminence-based medicine which is basically experience driven and more towards evidence-based medicine which is obviously based on research studies.” (Female, 5 years post-residency). Many interviewees felt that critical appraisal skills were of even greater value to emergency physicians because of the large amount of medical knowledge that they were responsible to understand for clinical decision-making.

Although many attending physicians noted that they improved their critical appraisal skills after graduating residency, they expressed concern that if these skills were not encouraged, residents may not practice their critical appraisal skills as attendings. “I certainly spent more time learning critical appraisal as an attending than I did during residency. But if you didn’t learn it during residency and you end up in sort of a community practice, the chances of you ever getting a chance to meaningfully do that again are small.” (Male, 20 years post-residency).

Residents felt that learning critical appraisal skills was valuable for their training by helping them to provide better patient care, and also felt that these skills would benefit them in the future when they would be responsible for their own continued medical education.

3. Motivation for critical appraisal

Most physicians noted both extrinsic and intrinsic motivating factors for learning critical appraisal, with intrinsic motivating factors perceived as having a bigger and more lasting influence overall.

### Extrinsic motivation

Many attending physicians noted that they were required to do a research project during residency. This assigned work forced them to become better at critical appraisal in order to complete the requirement. One physician held very little interest in critical appraisal until she had to do it as part of her research project.

“But I still didn’t care, you know, all that much and – where I really learned it was midway through residency I had to do a research project, and my project was, you know an original study that I designed that I got the IRB for … ultimately my article got published in a journal, so I had to actually do it for real, and it made me appreciate it in such a … more real way where I actually had to do it on my own...Because I’m very invested in something that’s my own project, so that’s when I actually forced myself to do it.” (Female, 2 years post-residency).

Although many physicians did not express enthusiasm for performing critical appraisal as a resident, the initial assigned research project allowed them to understand critical appraisal on a more meaningful level.

### Integrated regulation and intrinsic motivation

Internalized and intrinsic motivating factors were described by all attending physicians as contributing to a greater interest in critical appraisal. Most physicians described a desire to learn critical appraisal skills in order to provide better patient care and to keep up to date on current medical literature. Physicians also felt greater internalized motivation to understand critical appraisal in order to justify their clinical decisions once they became attendings who needed to teach residents.

4. Facilitating engagement with critical appraisal

Attending physicians and residents emphasized the importance of finding ways to make critical appraisal skills more relevant to residents. Distinct from practical teaching strategies, emergency physicians noted three major ways to support residents’ engagement with critical appraisal: normalization of critical appraisal skills within the ED culture, connection of critical appraisal skills to patient care, and connection of critical appraisal skills to resident research.

Almost all physicians emphasized the importance of promoting critical appraisal within the culture of the ED. Attending physicians noted that the ED where they trained and their attending mentors held a major role in shaping both their interest in critical appraisal and how likely they were to continue using it.

“But you have to normalize it as an expectation, meaning that the senior residents and the attending staff role model critical appraisal in their teaching and on shift and in other places … You have to make it feel not so much like a task but just like a normal part of the culture.” (Male, 20 years post-residency).

Emergency physicians also noted the importance of engaging residents in critical appraisal by relating it to patient care. A few physicians also felt that residents could become more engaged in critical appraisal by providing them with greater opportunities for meaningful research.

5. Teaching critical appraisal

In terms of the practical aspects of teaching critical appraisal, attending physicians noted the importance of thinking about critical appraisal as a tool or skillset rather than a topic.

“You have to sit down and learn critical appraisal separate from conjunctivitis or myocardial infarction. It should be a tool. It shouldn’t be a topic itself.” (Male, 20 years post-residency).

One physician felt that critical appraisal could be seen as similar to a language. Most emergency physicians should learn the basics in order to understand how to interpret medical literature, but very few physicians needed to become truly ‘fluent’ in critical appraisal. “You should have, like, a relatively constrained, unambitious set of skills that – sort of a curriculum. I think it should be a relatively small curriculum that focuses on the basics so that there’s at least some fluency with the language and people can understand, you know, what people are – what methodologists are even talking about when they say things like cohort study or a p-value, but often our critical appraisal curriculum is overambitious.” (Male, 12 years post-residency).

Many physicians noted the importance of incorporating medical literature into residency conference with an emphasis on performing critical appraisal frequently. Many physicians felt that the best way to have residents learn critical appraisal skills was to make the resident teach critical appraisal or be in charge of their own projects. Residents also felt that being in charge of projects involving critical appraisal or being asked to teach critical appraisal themselves would enhance their learning.

6. Level of competency with critical appraisal.

Physicians in general felt that residents should have a basic foundation in critical appraisal skills which they could continue to develop over their careers.

“I think we should all have a sort of a foundation and that we should have a specialized subset of emergency physicians and other methodologists who can tell the rest of us which studies are good and which studies are bad and what we should take from various studies …” (Male, 12 years post-residency).

Nearly everyone interviewed, however, from residents to the ED chair, felt that they could be better at critical appraisal.

## Discussion

Upon reviewing the current literature, to the best of the author’s knowledge, this is the first study exploring emergency physicians’ perceptions of critical appraisal skills. The results of this study highlight the challenges of learning critical appraisal as well as potential strategies to improve resident engagement in critical appraisal. Emergency physicians, especially during residency, described little initial interest in learning critical appraisal because it seems boring as well as difficult. Given the time constraints that residents face, in order to learn critical appraisal, it has to be integrated into the curriculum in a way that connects it to concepts that EM residents are more intrinsically motivated to learn and that limits excessive cognitive load [13, 15].

Intrinsic motivation can be defined as “a state that causes free engagement in an activity out of interest or for inherent satisfaction.” Although intrinsic motivation in theory can be distinguished from the most advanced form of extrinsic motivation, integrated regulation, in actual practice this is not a meaningful distinction [13]. This progression from an extrinsically motivated mindset to an intrinsically motivated one is critical in order to meaningfully train residents in critical appraisal skills.

In accordance with self-determination theory, residents are more inherently interested in learning about patient care. By demonstrating the value of critical appraisal skills in assisting emergency physicians with patient care decisions, residents will naturally build a greater interest in learning these skills. However, it is also important that residents are taught critical appraisal as a tool to help them learn their core EM knowledge, as opposed to a separate topic that can be perceived as creating extra work [15].

To promote residents’ interest in critical appraisal, it must be an integrated part of the ED culture. The importance of culture in shaping resident learning is described by social cognitive theory, in which much of human learning comes from observing and following the actions of role models in one’s immediate environment [18]. Journal clubs can be optimized to promote a culture of critical appraisal by teaching these skills in a structured and positive way with faculty role models to provide guidance. The more that attending physicians use critical appraisal in their clinical practice and incorporate it into residency conference and other residency activities, the more that residents will see critical appraisal as a valuable part of their education.

Upon reflection of the development of their own appreciation for critical appraisal, physicians often noted both extrinsic and intrinsic motivating factors. Given critical appraisal’s reputation for being uninteresting, a few required critical appraisal assignments may be needed to motivate residents, at least initially, to learn these skills. As physicians described in their interviews, the initial extrinsic motivation of an assigned research project often led to a deeper interest in critical appraisal when they felt they had a real stake in the project. Making residents responsible for meaningful projects requiring critical appraisal skills aligns with concepts of autonomy and competence described in self-determination theory. Residents who are in charge of their own learning but are still supported via mentorship will develop greater intrinsic motivation to learn critical appraisal, shifting from a purely extrinsic motivation mindset (research requirement) to a more enduring intrinsic motivation mindset (life-long practice of critical appraisal) [13, 19].

Although both residents and attending physicians were intrinsically motivated to learn critical appraisal in order to provide better patient care, physicians noted increasing intrinsic motivation once they became attendings so that they could perform well in one of their advanced professional roles (educator, researcher, etc.) In order to improve learning of critical appraisal, efforts to promote the development of professional identity should occur earlier in residency training. As residents start to see themselves not only as clinicians who value quality patient care, but also as teachers, researchers, or promoters of patient safety, they will intrinsically value critical appraisal more. Cruess et al. argue that medical education should focus not simply on teaching professionalism, but in the larger goal of professional identity formation. With dedicated mentorship, role-modeling, and explicit teaching about professional identity formation, trainees will be better supported in becoming the types of physicians they wish to be [20, 21].

**Fig. 2:**
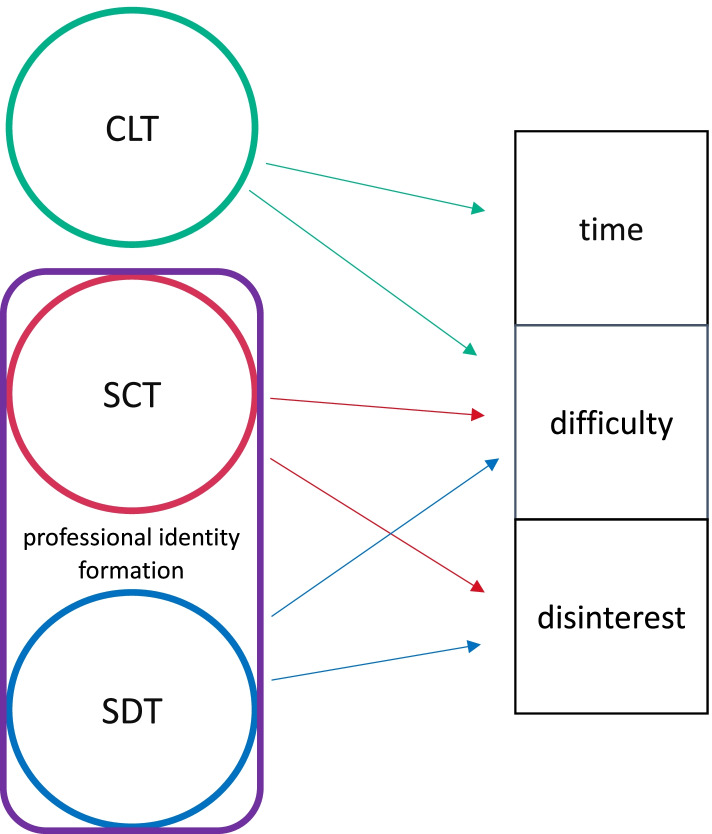
A unified approach to teaching critical appraisal skills. Concepts from CLT can be used to reduce the barriers of time and difficulty associated with the development of critical appraisal skills. Principles from SCT and SDT, along with the encouragement of professional identity formation, will help improve residents’ interest in critical appraisal and reduce perceptions of difficulty associated with critical appraisal skills

Based upon the different factors discussed, a unified approach to teaching critical appraisal skills (Fig. 2) can provide a framework for EM residency programs which could optimize learning of critical appraisal skills. Components of CLT can help reduce extraneous cognitive load, mitigating the barriers of time and perceived difficulty associated with learning critical appraisal. SCT can be used to improve residents’ interest in critical appraisal and help reduce the perception that it is difficult, as residents will naturally begin to integrate critical appraisal skills into their practice once it becomes part of the ED culture. Concepts from SDT can also be used to build residents’ intrinsic motivation for critical appraisal. Lastly, an explicit focus on encouraging residents’ professional identity formation, which combines components of self-efficacy, autonomy and community emphasized in SCT and SDT, will further promote residents’ interest in critical appraisal.

### Limitations

The study’s major limitation is the inclusion of only one specialty at one institution, which may affect the generalizability of the findings [22]. It is not clear if physicians in specialties other than emergency medicine would describe similar challenges with learning critical appraisal, and if the proposed teaching strategies would similarly improve all residents’ motivation to learn critical appraisal. Given that this study only included physicians who work at an academic medical center, the findings described in this study may not be applicable to physicians at community hospitals or in different countries where institutional practice and expectations in regards to critical appraisal may be different. Future studies could compare physicians’ perceptions of critical appraisal in different hospital environments as well as across specialties and countries to improve transferability [23].

## Conclusion

Using a grounded theory approach, this study provides a detailed analysis of the challenges as well as the benefits of learning critical appraisal skills, and how these skills can best be taught to EM residents [24]. Key barriers to learning critical appraisal include time constraints and the perception that critical appraisal is difficult or boring. By integrating critical appraisal into ED culture and connecting critical appraisal skills with the development of residents’ professional identity as excellent physicians, researchers, and teachers, residents will develop greater intrinsic motivation to learn and practice critical appraisal.

## Supplementary Information

**Additional file 1:** Appendix A.

**Additional file 2:** Appendix B.

## Data Availability

The datasets analyzed during the study are available from the corresponding author on request.
