# Supplementary material for: Emergency physicians’ perceptions of critical appraisal skills: a qualitative study
Source: BMC Med Educ. 2022 Apr 15;22:287. doi: 10.1186/s12909-022-03358-y (PMC9013089; doi:10.1186/s12909-022-03358-y)
Supplement: Supplementary file 1 — Additional file 1: Appendix A. [file 12909_2022_3358_MOESM1_ESM.docx]

Appendix A

Interview Guide

1. How do you define critical appraisal of the medical literature?

2. How knowledgeable do you consider yourself to be about critical appraisal?

3. Why do you think critical appraisal is (or is not) useful to emergency physicians?

4. How were you taught critical appraisal during your training (medical school, residency) i.e. journal clubs, lectures, etc.?

5. What do you consider the main barriers to learning critical appraisal skills?

5a. Why?

6. How do you think we should teach critical appraisal skills to trainees despite these barriers?

7. As a resident, what motivated you to learn critical appraisal skills?

8. In your opinion, what factors would promote learning of critical appraisal among residents?

9. How often do you perform critical appraisal now?

10. What motivates you to perform critical appraisal now?

10. How often do you read journal articles or critically appraise other current medical resources?

12. How skilled should the average emergency physician be in critical appraisal? Should there be different standards for academic vs community EPs?

13. What do you think the role of critical appraisal is now that we obtain a lot of our medical information online, and when so much medical opinion is available on social media?
