# Supplementary material for: Emergency physicians’ perceptions of critical appraisal skills: a qualitative study
Source: BMC Med Educ. 2022 Apr 15;22:287. doi: 10.1186/s12909-022-03358-y (PMC9013089; doi:10.1186/s12909-022-03358-y)
Supplement: Supplementary file 2 — Additional file 2: Appendix B. [file 12909_2022_3358_MOESM2_ESM.docx]

**Appendix B**

| Themes | Codes | Definition | Quotes |
| --- | --- | --- | --- |
| Barriers to critical appraisal | Lack of interest | Disinterest in learning critical appraisal | - “A dry topic and doesn’t engage the learners, especially someone who is young…” - “Boredom. And I think it’s because maybe it just suffers from a lack of context, right?” - “So I think there’s a level of it not being the most compelling topic in the world.” - “Yeah, so for physicians, especially emergency physicians, is that they’re not interested.” - “It is so different from all the other things that we need to know. I think people probably see it as less important because there’s no physiological outcome based on it and there’s no – we live in sort of like [an] outcome-oriented world, does the patient feel better, is the patient dead...This is seemingly less important.” - “I mean, I’m barely interested in research now, and I think back then, I was really uninterested in research, and so it was a lot of terms that applied to a subject that I barely cared about. And so it was really hard, I think, to listen to them and incorporate them in your brain in a way that made it stick because it just wasn’t as fascinating as, you know, listening to how to manage, you know, trauma patients or something like that, right?” |
|  | Time limitations | Time constraints that impede learning of critical appraisal | - “Time…Competing interests with other things that they want to do, competing interests with other things in the curriculum.” - “I think it’s hard especially as a resident to just be focusing on even just learning the literature.” - “You have a lot of stuff to learn in a relatively short period of time and the volume of information.” - “Being a resident, like, we’re so busy already to begin with. It’s just hard to find the extra time to, like read a journal article.” - “That time dedicated to reading, like, the newest literature Is maybe better spent, like, making sure you have a good foundational knowledge of everything we need to know in our specialty.” - “I think that all skills have to compete with each other as far as time inside of a curriculum.” - “I have two children, and I tend to not add extra tasks unless I have to.” |
|  | Difficulty | Perception that critical appraisal is too complex/challenging | - “Most people don’t like doing things they’re not good at, right.” - “I think it is hard to know what you don’t know if you don’t know it." - “So I think it sometimes feels like you won’t ever reach that expert level so you just kind of give up.” - “But if you’re kind of newer to it, then if you’re reading the methods, like a lot of times the methods are really difficult to actually parse out what they’re saying, so that’s a barrier.” - “There’s just too much out there and there’s too many people competing to be the guru of EBM…It used to be you go to the library and there was this book. It’s overwhelming. How do you tease that out? …So then people can shut down. Like, oh, I don’t know. I don’t know. This says this, this says this, so whatever.” - “So I guess there are many new articles coming out in many, many journals, and it’s a little bit overwhelming, I think, for medical professionals to even attempt to sort through it.” |
| Engagement | Engagement through patient care | Enhancing residents’ interest in critical appraisal by making connections with patient care | - “So I think if there is a way to link it back to, this is what you can use on shift, this is what can impact your patients, this is how you can take this information and actually use it on a day-to-day basis, would help a lot.” - “So you know, how do you make these things seem relevant to them? And I think that maybe, you know ,you have to put them in a position, I think, where they are actually using it in a day-to-day basis to do their basic work…you have to give them the opportunity to incorporate the literature into their practice on a day-to-day basis and the skills that they need to do it well.” |
|  | Engagement through research/projects | Enhancing residents’ interest in critical appraisal through research/projects | - “I think, you know, the easiest way would be to get them involved in projects where the skills that you need for critical appraisal of the literature were, you know, were actually being used or developed.” |
|  | Engagement through normalization | Enhancing residents’ interest in critical appraisal by making it part of the culture/environment | - “I think in some ways it should be just a little more integrated…I think you just have to normalize it.” - “But you have to normalize it as an expectation, meaning that the senior residents and the attending staff role model critical appraisal in their teaching and on shift and in other places…You have to make it feel not so much like a task but just like a normal part of the culture.” - “I think the culture of the place where you worked, you know, sort of it’s a place where attendings are citing papers, critiquing papers and during the shift sort of making it – integrating it into everyday clinical practice…” - “Even if you don’t love it, you don’t – you’re not completely sold on it, you’ve done it enough times where you can do it if you have to do it. And so then that’s just consistency…” - “I think it’s just being more familiar with it and doing it enough times that it becomes more second nature and doesn’t take as much mental effort to go through.” (resident) |
| Motivation | Intrinsic Motivation | Inherent desire or curiosity to learn | - “I didn’t have any intrinsic motivation until after I graduated and I had to teach residents. That’s actually what intrinsically motivated me because I felt like I had to have good reasons for why I was practicing the way that I was.” - “Interesting cases that bring up grey areas of thought and approach.” - “It’s patients and questions that come up on shifts and then if I’m ever trying to prepare a discussion for, like, a small group learning session, I’ll go over something in that setting.” - “I was very displeased to it so I took upon myself to start reading, start educating myself and start actual appraising literature the way I…would have never done otherwise had I not been displeased with the way that my supervisor and attendings were doing things.” - “I will tell you that after residency, I really learned it. So it’s more of a self-learning thing afterward sort of forced by working in an academic environment.” - “To be completely honest with you, there was a resident who was in my class who was very well read, and I got sort of competitive with her. That’s kind of how I got into it was just trying to keep up with her.” - “I think a lot of it is habit. I formed this habit. I’m just like a very habitual person, and it’s sort of like become like a meme.” - “Trying to figure out what is the best way to manage my patients.” (Resident) - “My biggest motivator has been, like, probably my teachers because….certain things that have been accepted for such a long time, and then, you know, someone writing an article about, like, how this might not be the best practice. Having my teachers, like, point that out to me kind of, like makes me a little bit more curious about, like, the whole process.” (Resident) - “I am going to be an attending next year, or, like, at least, a fellow, and I will be teaching. Like, that’s another motivator to…keep on top of new things that are coming out so that I know what to teach my residents.” (Resident) - “Being able to have sound reasoning for why I’m doing something or not doing something is really helpful in the teaching aspect of emergency medicine. And so I am motivated because I don’t want to sound stupid when I am teaching.” (Resident) - “I think, you know, being in emergency medicine, you’re exposed to a lot of different – you need to know a little bit about a lot of different things, and having sound – a sound kind of footing to stand on with your decision-making process is something that I think we all strive for, whether or not we’re conscious about it. And I think critical – or going through critical appraisal is – of the literature is one way to kind of solidify that.” (Resident) |
|  | Extrinsic Motivation | External regulation of learning | - “But I still didn’t care, you know, all that much and – but then what – where I really learned it was midway through residency I had to do a research project, and my project was, you know an original study that I designed that I go the IRB for…And then, you know, ultimately my article got published in a journal, so I had to actually do it for real, and it made me appreciate it in such a – like, such a more real way where I actually had to do it on my own...Because I’m very invested in something that’s my own project, so that’s when I actually forced myself to do it.” - “And each resident had a research requirement…So but at where I was, you had a mentor. You worked closely with that mentor. There was a research methodology component to your research project, and there was just a much higher expectation that you would learn how to do – you would learn a lot about the study that you are trying to accomplish, and I did.” - “There were definitely lectures on it. Every month, we would have some sort of general discussion where we were expected to apply our sort of review of – like, I guess do, like a critical analysis of the paper…So we did a lot, and I will also add in that it was not my favorite part of what we had to do.” |
| Practical teaching of critical appraisal | Teaching critical appraisal | Specific strategies and concepts for teaching critical appraisal skills | - “The way to learn is by teaching or facilitating.” - “I wonder if you could get a shift where you’re not really seeing patients…where you could bring up a patient and a case. We’re doing more resident presented journal club, which I think is good where they’re putting the impetus on us to at least try because I think really [that] you know when you teach it hopefully…” - “It’s the kind of thing that has to be incorporated longitudinally and it should be incorporated continuously. I would incorporate some critical appraisal primary sources in the conference on a weekly basis.” - “You have to sit down and learn critical appraisal separate from conjunctivitis or myocardial infarction. It should be a tool. It shouldn’t be a topic itself.” - “Making it part of the regular curriculum. You do it enough times that already know what the – you anticipate the next steps and you’re already thinking about that kind of stuff, and it doesn’t take so much active mental energy. And so I think reducing the cognitive load involved with critical appraisal is how to make it happen more often, and making it happen more often is how you, like, make it more widely used in EM.” (resident) |
| Importance of Critical Appraisal | Importance of critical appraisal to specialty | Value of critical appraisal for the field of emergency medicine | - “Literature is what guides our practice, right? So it’s also important to be able to read to figure out, like, you know, what’re real good evidence and what’s bad evidence and, you know, and that’s going to affect our patients down the line.” - “I also use critical appraisal in teaching patients.” - “So I think that, you know, that we all, as doctors, have a responsibility to contribute to the body of knowledge of medicine, but I think in some ways emergency medicine physicians have a greater responsibility because we are, at times, maybe every day by our consultants, viewed as maybe less knowledgeable or less scholarly or even less through and complete in the way that we care for our patients. So if you care about the way, you know, you’re viewed individually and your specialty is viewed as a whole, I think that, you know, we probably not just have to be good at it, but in some ways, we have to be better than our colleagues that way we can be more respected.” - “For the betterment of our patients and the system as a whole, then we have to be a little bit more careful about the information that we utilize and practice.” |
|  | Importance of critical appraisal for residents | Value of critical appraisal for residency training | - “I think it would benefit the specialty or benefit the graduates if we were better at providing more critical appraisal time for residents in training…I think from a long-term practice perspective, the more you get people early in the course of their training, the better off they’ll be.” - “I think it really helps when you’re advocating for your patient with a consultant often times to be able to say with [like] evidence behind your statements.” (Resident) - “I still think it’s a vital part of the process of developing your own learning system, especially, for I imagine, post-residency where you’re – it’s going to be self-directed and self-guided learning, and building the critical appraisal skills, that is something that is probably vital to maintaining a robust self-education system.” (Resident) |
| Level of competency with critical appraisal | Level of critical appraisal skills | Degree of competency that emergency physicians should have with critical appraisal | - “The goal is they should be able to read a paper and be able to interpret the results in a context that allows them to apply it directly to patient care.” - “I think everybody can be better at figuring out how to critically appraise the literature. I don’t know that there’s a good, clear external criteria standard for what constitutes competency or lack of competency in that regard.” - “I think they should be comfortable being able to read an article and saying to themselves, is this a legit study? And whether or not that’s going to affect their practice?” |
